# Comparison of B-Cell Lupus and Lymphoma Using a Novel Immune Imbalance Transcriptomics Algorithm Reveals Potential Therapeutic Targets

**DOI:** 10.3390/genes15091215

**Published:** 2024-09-17

**Authors:** Naomi Rapier-Sharman, Sehi Kim, Madelyn Mudrow, Michael T. Told, Lane Fischer, Liesl Fawson, Joseph Parry, Brian D. Poole, Kim L. O’Neill, Stephen R. Piccolo, Brett E. Pickett

**Affiliations:** 1 Department of Microbiology and Molecular Biology, Brigham Young University, Provo, UT 84602, USA; naomi.rapier.sharman@gmail.com (N.R.-S.); mudrowm@byu.edu (M.M.); brian_poole@byu.edu (B.D.P.); kim_oneill@byu.edu (K.L.O.); 2 McKay School of Education, Brigham Young University, Provo, UT 84602, USA; lane_fischer@byu.edu; 3 Department of Statistics, Brigham Young University, Provo, UT 84602, USA; lieslfawson@gmail.com; 4 Department of Comparative Arts and Letters, Brigham Young University, Provo, UT 84602, USA; joseph_parry@byu.edu; 5 Department of Biology, Brigham Young University, Provo, UT 84602, USA; stephen_piccolo@byu.edu

**Keywords:** immune imbalance, immune imbalance transcriptomics (IIT), B-cell lymphoma, systemic lupus erythematosus (SLE), RNA-Seq, algorithm, drug discovery, cancer, autoimmune disease

## Abstract

Background/Objectives: Systemic lupus erythematosus (lupus) and B-cell lymphoma (lymphoma) co-occur at higher-than-expected rates and primarily depend on B cells for their pathology. These observations implicate shared inflammation-related B cell molecular mechanisms as a potential cause of co-occurrence. Methods: We consequently implemented a novel Immune Imbalance Transcriptomics (IIT) algorithm and applied IIT to lupus, lymphoma, and healthy B cell RNA-sequencing (RNA-seq) data to find shared and contrasting mechanisms that are potential therapeutic targets. Results: We observed 7143 significantly dysregulated genes in both lupus and lymphoma. Of those genes, we found 5137 to have a significant immune imbalance, defined as a significant dysregulation by both diseases, as analyzed by IIT. Gene Ontology (GO) term and pathway enrichment of the IIT genes yielded immune-related “Neutrophil Degranulation” and “Adaptive Immune System”, which validates that the IIT algorithm isolates biologically relevant genes in immunity and inflammation. We found that 344 IIT gene products are known targets for established and/or repurposed drugs. Among our results, we found 48 known and 296 novel lupus targets, along with 151 known and 193 novel lymphoma targets. Known disease drug targets in our IIT results further validate that IIT isolates genes with disease-relevant mechanisms. Conclusions: We anticipate the IIT algorithm, together with the shared and contrasting gene mechanisms uncovered here, will contribute to the development of immune-related therapeutic options for lupus and lymphoma patients.

## 1. Introduction

Systemic lupus erythematosus (lupus) is a debilitating autoimmune disease with widely varying clinical manifestations, affecting an estimated 3.41 million people [1,2]. The pathology of lupus is perpetrated in part by autoreactive B cells, which produce auto-antibodies against self-antigens such as DNA, nuclear proteins, and other damage-associated molecular patterns (DAMPs) that remain when cells are damaged or undergo apoptosis [3]. Lupus has a complex diagnostic process, and many patients wait for years to receive a valid diagnosis. Lupus also has a substantial impact on the daily life of patients and their families due to severe symptoms (kidney failure, severe rash, depression, chronic fatigue, chronic pain, etc.) and limited treatment options, sometimes with undesirable side effects. Hydroxychloroquine, glucocorticosteroids, and immunosuppressive drugs are effective at attenuating lupus symptoms and have been the standard of treatment for decades, with the recent addition of biologics such as belimumab. Hydroxychloroquine is still considered the cornerstone of most lupus treatments, despite the long-term risk of retinal toxicity, which can contribute to subsequent patient blindness [4]. Glucocorticoids are impressively effective at treating lupus symptoms in the short term but can cause permanent damage to multiple organ systems, cataracts, osteoporosis, and coronary artery disease after long-term use or at higher-than-standard dosing [5,6,7].

Lymphomas are estimated to make up 5% of malignancies worldwide [8], with ~450,000 new cases of non-Hodgkin’s lymphoma annually, leading to 240,000 deaths per year [9]. In addition, up to 90% of non-Hodgkin’s lymphomas originate from B cells [10]. Many B-cell non-Hodgkin’s lymphomas (lymphomas), including diffuse large B-cell lymphoma, are aggressive and difficult to treat without severe side effects [8]. The slower-moving indolent lymphomas are typically considered incurable and, while patients are subjected to milder treatments such as rituximab–bendamustine (R-Benda), they run the long-term risk of cumulative toxicity from repeated chemotherapies and tumor progression into an aggressive lymphoma subtype [8]. The first-line therapy for fast-moving lymphomas is aggressive combination chemotherapy, such as R-CHOP (rituximab, cyclophosphamide, vincristine, and prednisone). In the case of relapse, the first-line treatments are followed by anti-CD19 chimeric antigen receptor (CAR) T-cell therapy or more intense chemotherapy regimens, which all require inpatient hospital stays, such as R-ICE (rituximab, ifosfamide, carboplatin, etoposide) or high-dose chemotherapy with autologous stem cell rescue (HD-SCT) regimens such as the Nordic protocol (administration of maxi R-CHOP, alternating with rituximab and high-dose cytarabine) [8]. Though some sources estimate lymphoma patient survival to be as much as 72%, the price paid to reach survival takes a heavy toll on the body and on the patient’s quality of life as they deal with rampant toxicity from chemotherapies, B-cell aplasia, or being immunocompromised from currently approved anti-CD19 CAR T-cell therapies [8,11].

Patients with lupus are seven times more likely to develop B-cell lymphomas than healthy patients, suggesting a possible mechanistic relationship between the two diseases [12]. An important aspect of both lupus and lymphoma progression, and of their relationship, is immune involvement. The immune system is generally responsible for tolerating self-tissue, for removing non-self threats (including cancers, bacteria, and viruses), and for coordinating the healing of damaged self-tissue following the removal of non-self threats. Healthy immune function begins and ends with a non-inflamed homeostasis, which could be termed as ‘immune balance’ [13,14]. Immune activation against threats is primarily accomplished via inflammatory signaling (including but not limited to eicosanoids including B- and E-prostaglandins and leukotrienes, vasoactive amines, complement cascade, kinins and, depending on the circumstances, cytokines such as Tumor Necrosis Factor α (TNFA), Interferon γ (IFNG), Interleukin 1 (IL1), Interleukin 2 (IL2), Interleukin 6 (IL6), Interleukin 12 (IL12), Interleukin 17 (IL17), Interleukin 18 (IL18), and Interleukin 23 (IL23)) [15]. In contrast, immune tolerance of self-tissue and wound healing/cleanup are accomplished via anti-inflammatory/pro-resolving signaling (including but not limited to nitric oxide, adenosine, cortisol, steroids, eicosanoids including lipoxins, resolvins, and D-prostaglandins, protectins, Interleukin 10 (IL10), and Transforming Growth Factor β 1 (TGFB1)) [16]. Conditions such as cancer or autoimmune diseases develop when this immune balance is disrupted by a failure of immune detection and activation (cancer), or a failure of immune tolerance and resolution (autoimmune disease). Intense inflammation associated with immune activation from several causes (infection, allergy, trauma, etc.) can set the stage for autoimmune development [17,18]. Failure to remove inflammation-inducing, damage-associated molecular patterns (DAMPs) and/or pathogen-associated molecular patterns (PAMPs) can lead to the loss of self-tolerance [19]. In particular, pathogen molecular mimicry and the creation of cross-reactive and/or DAMP-targeted antibodies (e.g., anti-DNA) are known mechanisms of autoimmune disease development [17,18]. The failure of immune tolerance is integral to autoimmune development. In contrast, the inability of the immune system to effectively identify and eliminate tumor cells can result in immune tolerance that enables the growth and progression of cancer [20]. Cancer develops as tumor cells create subclones and strive to avoid immune surveillance via immune-silencing strategies such as the Programmed Cell Death 1 (PD1)/Programmed Cell Death 1 Ligand 1 (PDL1) blockade [21,22]. Cancer development is dependent upon high immune tolerance of the tumor, which develops via natural selection as non-immune-avoidant cancer cells are killed and successful immune-evading cancer subclones proliferate [20,22]. The failure of sustained immune activation is integral to cancer development. Though there is some contrast suggested between lupus and lymphoma, lupus and lymphoma depend on an imbalance of immune function. This similarity may contribute to the co-occurrence of lupus and lymphoma.

An additional advantage to the approach of comparing a cancer and an autoimmune disease from the same tissue type is the possible outcome of targeted therapies that harness the contrasting mechanisms of disease. It is well known that tumors can be treated by inducing cancer-specific autoimmunity [23,24], suggesting that, to some degree, cancers and autoimmune diseases can be regulated by contrasting mechanisms. This contrast is seen by the apparent opposite utilization of the following two complementary aspects of the immune system: immune activation (autoimmunity) and immune tolerance (cancer) [13,14]. Appropriate immune homeostasis, which is characterized by proper immune activation and subsequent resolution, is not present in either autoimmune disease (failure to tolerate self and resolve inflammation) or cancer (failure to activate sufficiently to detect and clear non-self-tumor) [15,16]. Some of the most apparent examples of the “opposite mechanisms” at play in cancer and autoimmune disease involve immunotherapies (steroids, T-cell therapies, checkpoint inhibitors, other monoclonal antibody therapies, oncolytic viruses, etc.) [25,26]. The growing utilization of immunotherapies in cancer has revealed a fascinating paradox—the rare occurrence of immune-related adverse events, specifically, a loss of self-tolerance leading to autoimmunity [25,26]. The occurrence of cancer immunotherapy-related adverse immune events, such as cytokine storms (and other immune-related inflammatory phenomena) or the induction of tissue-specific or systemic autoimmunity, point to a new and largely untouched area of research regarding the relationship between cancer and autoimmunity and what factors determine whether adverse immune events develop in a patient treated with immunotherapy [27]. Conversely, some patients do not respond strongly enough to cancer immunotherapies to clear or inhibit tumor growth [28]. In autoimmunity, anti-inflammatory immunotherapies may potently improve the patient’s quality of life but can also increase the risk of infection. The immunotherapeutic silencing of immune activation can also contribute to the development of cancer in autoimmune patients who are treated with anti-inflammatory drugs for long periods [7]. Though successful immunotherapies and other therapeutics that modulate immune function are seminal achievements of substantial research efforts in the fields of cancer and autoimmunity, the failure of some patients to return to healthy immune homeostasis demonstrates that there is still room for development.

Despite the higher-than-expected rate of lupus and lymphoma co-occurrence, and the possibility of utilizing contrasting gene mechanisms to strengthen lupus and lymphoma immunotherapies, studies that directly compare and contrast lupus and lymphoma gene expression using bulk RNA-sequencing are rare. Additionally, there are no datasets in the National Center for Biotechnology Information (NCBI) Gene Expression Omnibus (GEO) database that include bulk or single-cell RNA sequencing (RNA-seq) samples from patients with both conditions in the same study [29].

This lack of available datasets containing lupus, lymphoma, and healthy B cell RNA-seq data compelled us to combine the results from multiple public experiments to facilitate a secondary analysis of human RNA-seq data. As such, the current study examined the existing gene expression data from publicly available datasets in an attempt to understand the mechanistic transcriptional similarities and differences between these two conditions. We then used these RNA-seq results as a use-case for our novel Immune Imbalance Transcriptomics (IIT) algorithm. This algorithm aims to provide a reproducible workflow to determine the top gene candidates that best represent the immune imbalance for a consistent tissue type (e.g., B cells). We anticipate that identifying the shared and contrasting underlying mechanisms between these two contrasting B-cell-related diseases can be used to determine the relevant mechanisms that contribute to immune imbalance and can therefore be used in the identification of candidate targets and therapeutics that can be developed or repurposed to improve outcomes for these diseases.

## 2. Materials and Methods

Public RNA-sequencing datasets from the GEO database [29], originally generated from clinical material, were manually screened to find primary B-cell lymphoma, B-cell lupus, and healthy B-cell control samples. As previously performed [30], B-cell lymphoma RNA-sequencing samples were acquired using the search term “B-cell lymphoma”, with the goal of finding B-cell non-Hodgkin’s lymphoma (lymphoma) samples and healthy B-cell controls. All 208 studies matching the phrase “B-cell lymphoma” were manually reviewed. All 34 studies matching the phrase “(B-cell SLE) OR (B-cell lupus)” were also manually reviewed. In addition, the top 238 of 842 studies retrieved using the “(B-cell) NOT ‘cell line’” query were manually reviewed to increase the number of B-cell healthy control samples. Specifically, before the manual review, the query results were automatically reduced using the built-in GEO filters “Homo sapiens” to include only samples collected from humans and “Expression profiling by high-throughput sequencing” to include only RNA-seq datasets. To minimize the downstream impact of irrelevant data, RNA-seq samples from cell lines, formalin-fixed paraffin-embedded tissues, gene expression microarray experiments, single-cell RNA-sequencing experiments, xenografts, metastases, adjacent normal tissues, sequencing performed on platforms other than Illumina, samples known to be infected (Epstein-Barr virus (EBV), Kaposi’s sarcoma-associated herpesvirus (KSHV), Human Immunodeficiency Virus (HIV)), and samples which contained more diverse tissue types (i.e., whole blood, peripheral blood mononuclear cells (PBMCs), brain, etc.) were manually identified and excluded. While a subset of the healthy control samples was obtained from the same studies as the cancer samples, other healthy control samples were obtained from other studies to create roughly equivalent-sized cancer and healthy groups. The final dataset that was assembled for secondary analysis consisted of 361 samples (135 lymphoma samples, 138 lupus samples, and 89 healthy B-cell samples) from 16 studies (Table 1) [31,32,33,34,35,36,37,38,39,40,41,42,43,44,45].

### 2.1. Preprocessing of RNA-Sequencing Data

Following the manual curation of the RNA-seq samples, the FASTQ sequencing files were pre-processed as previously described [30,46,47,48,49]. In brief, FASTQ files containing RNA-sequencing data were downloaded from the Sequence Read Archive (SRA) using the sra-tools software (version 2.10.8) [50]. The FASTQ files, the associated metadata file (Supplementary File S1), and a configuration file were used as inputs to the Automated Reproducible MOdular workflow for preprocessing and differential analysis of RNA-seq data (ARMOR) workflow (version 1.3.0) [51,52]. More specifically, the ARMOR Snakemake-based workflow includes the following steps: trimming sequencing adapters and poor-quality regions of the reads with TrimGalore! [53], generating quality control metrics with FastQC [54], mapping and quantifying reads to the human GRCh38 transcriptome with Salmon (version 1.3.0) [55], calculating significant differential gene expression using edgeR (version 4.0.16) [56], and performing Gene Ontology (GO) enrichment against terms from the Molecular Signatures Database (MSigDB) [57] using the Camera algorithm (version 3.50.3) [58]. Differentially expressed genes calculated by ARMOR were evaluated with log2 fold-change (log2FC) and false discovery rate (FDR)-adjusted *p*-values.

### 2.2. Principal Component Analysis

Following the mapping and quantification of the gene read counts with Salmon (version 1.3.0) [55], the transcript-level read counts were aggregated to each relevant gene, and the gene read counts were normalized per gene to control for outlier genes with high read counts before performing principal component analysis (PCA) to determine the relatedness of the included samples. PCA results were graphed using ggplot2 (version 3.5.0) [59] to visualize the disease groups and potential batch effects.

### 2.3. Immune Imbalance Determination

The IIT algorithm was then used to identify the shared and contrasting mechanism(s) that may include novel therapeutic targets across the disease states (lupus and lymphoma). In brief, the IIT algorithm (Supplementary File S2) reads the sample metadata and transcript-level read counts that are each produced by the ARMOR workflow (Supplementary Files S3 and S4) and runs differential gene expression analysis internally using edgeR (version 4.0.16) [56]. IIT uses the log2-fold-change (log2FC) and false discovery rate (FDR)-adjusted *p*-value data from one cancer vs. healthy (lymphoma vs. healthy) differentially expressed gene (DEG) comparison and one autoimmune vs. healthy (lupus vs. healthy) DEG comparison, all from the same tissue type (B cells). This enables the algorithm to effectively determine any shared or contrasting mechanisms between both comparisons that are relevant to the biology of the tissue type studied. To efficiently weight the fold-change values by the FDR *p*-values, the algorithm multiplies the log2FC values by the −log10FDR *p*-value for each gene, which increases the numerical value for highly significant results. To allow for an easier comparison, the (log2FC) × (−log10FDR *p*-value) products from both comparisons (lupus vs. healthy; lymphoma vs. healthy) were z-score normalized. All products were squared to remove negative values and then square-rooted to minimize the amplification of outliers, generating the equivalent of an absolute value. The square-rooted results from the two comparisons (lupus vs. healthy; lymphoma vs. healthy) were then summed together to yield an intermediate score metric for each gene. Ratios of the square-rooted results for each of the two preceding comparisons were computed to determine which comparison contributed more to the intermediate score metric for each gene. To appropriately weight the differentially expressed genes with similar intensity in both disease conditions (compared to healthy controls), the intermediate score metric was multiplied by the smallest of the ratios for each gene. This analysis’ resulting final immune imbalance scores were then stored in tabular form. 

To calculate a *p*-value for these final immune imbalance scores, the metadata labels (e.g., lymphoma, lupus, or healthy) were randomly permuted for all samples in the original datasets. This iterative permutation and re-calculation of immune imbalance scores was performed for 1000 replicates to create a null distribution of the immune imbalance scores. The entire null distribution of 18,486,468 IIT scores from 1000 permutations, as well as the original analysis, was normalized via z-score, and the *p*-values were calculated for the upper tail of the distribution. Following the *p*-value calculation, a Bonferroni adjustment was applied to correct for multiple hypothesis testing and reduce the likelihood of false positives [60]. Gene frequency counts were used to control for the batch effects in the significant IIT results in order to see how often each gene appeared in the portion of the null distribution that was IIT-significant. The frequency counts were then z-score normalized and the *p*-values were calculated for each gene frequency count. Based on this *p*-value, genes with significant gene frequency counts (*p* < 0.05) were removed from the significant IIT results to minimize the noise from the batch effects.

### 2.4. Immune Imbalance Validation

Enrichment analysis was then performed, using enrichR (version 3.2) [61,62,63] to identify the significant Gene Ontology (GO) terms and intracellular signaling pathways for these IIT results [64,65,66,67,68]. Enrichment results were visualized by quadrant using the ggVennDiagram (version 1.5.2) and UpSetR (version 1.4.0) packages [69,70]. GO terms were also compiled as supplementary material for each significant IIT gene result using the biomaRt (version 2.58.2) package [71,72]. 

Significant genes were then compared against the Open Targets Platform database (version 24.06) target–disease association profiles for “systemic lupus erythematosus” (EFO: MONDO_0007915) and “B-cell non-Hodgkins lymphoma” (EFO: EFO_1001938) [73]. Known targets for each disease were obtained by manually downloading the “Known Drugs” table from the Open Targets Platform (version 24.06) disease profile pages and extracting the gene identifiers associated with the targets hit by the drugs. This list was then compared to the IIT significant gene results to evaluate the presence of IIT results among the known drug targets. Results that overlapped between the significant IIT results and the known drug targets were sorted according to their IIT scores, with the highest-scoring IIT genes considered first.

## 3. Results

### 3.1. IIT Algorithm Design Solutions

**Figure 1:**
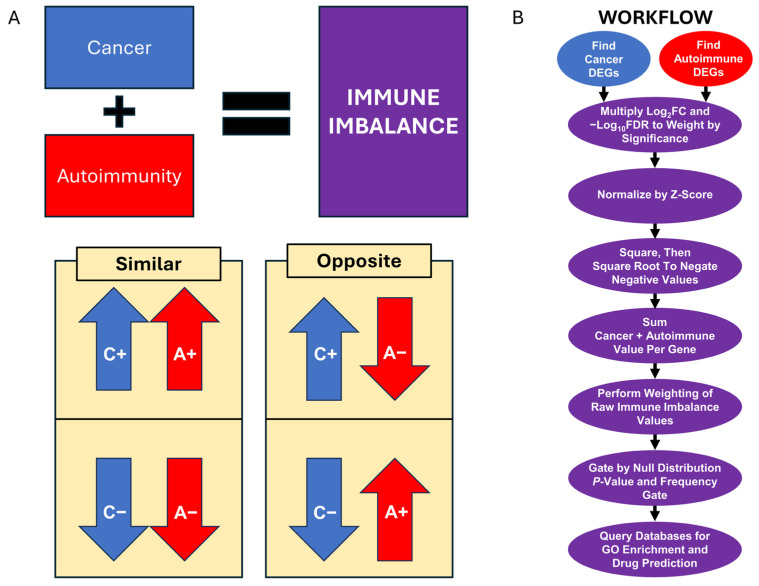
Rationale and Workflow Visualization for the Immune Imbalance Transcriptomics (IIT) algorithm. (**A**) Using cancer, autoimmune, and healthy control data from the same tissue type, IIT seeks to identify differentially expressed genes from four quadrant profiles, which can be described in two distinct groupings: (1) two profiles that both indicate similar gene expression, including genes upregulated in both cancer and autoimmunity (C+A+) and genes downregulated in both cancer and autoimmunity (C−A−); and (2) two profiles that show opposite gene expression, that is, genes upregulated in cancer but downregulated in autoimmunity (C+A−) and genes downregulated in cancer but upregulated in autoimmunity (C−A+). The results and discussion refer to these four immune imbalance quadrant profiles. (**B**) A visual representation of the IIT algorithm workflow (see Section 2, Materials and Methods for additional detail).

We designed the IIT algorithm with the purpose of identifying shared immune- and inflammation-related genes by comparing a cancer from the same tissue type to an autoimmune disease and to healthy tissue-specific controls (Figure 1A). Though immunotherapies have been a huge step forward in the treatment of both autoimmune diseases and cancers, the varying and sometimes unexpected outcomes of immunotherapy treatment indicate gaps in our current understanding of how the immune system works and which mechanisms are important to consider when manipulating immune function. Comparing cancer, autoimmune disease, and healthy samples from the same tissue type in this way should aid in identifying relevant and targetable immune- or disease-specific mechanisms.

To facilitate downstream comparisons between cancer and autoimmune disease, we deemed it important to first establish their relationships to a healthy baseline. As such, the first step of IIT was to calculate differential gene expression between an autoimmune/healthy pairing (in this case, lupus/healthy B cells) and a cancer/healthy pairing (B-cell lymphoma/healthy B cells). By comparing the lupus/healthy and lymphoma/healthy results, we can find differentially expressed genes with extreme log2-fold-change values (log2FCs) in both disease conditions as compared to the healthy baseline. Comparing cancer to autoimmune disease to study the immune system is a relatively new approach, with its own set of difficulties, each of which we addressed in the IIT workflow (Figure 1B) and which are explained below. 

**Figure 2:**
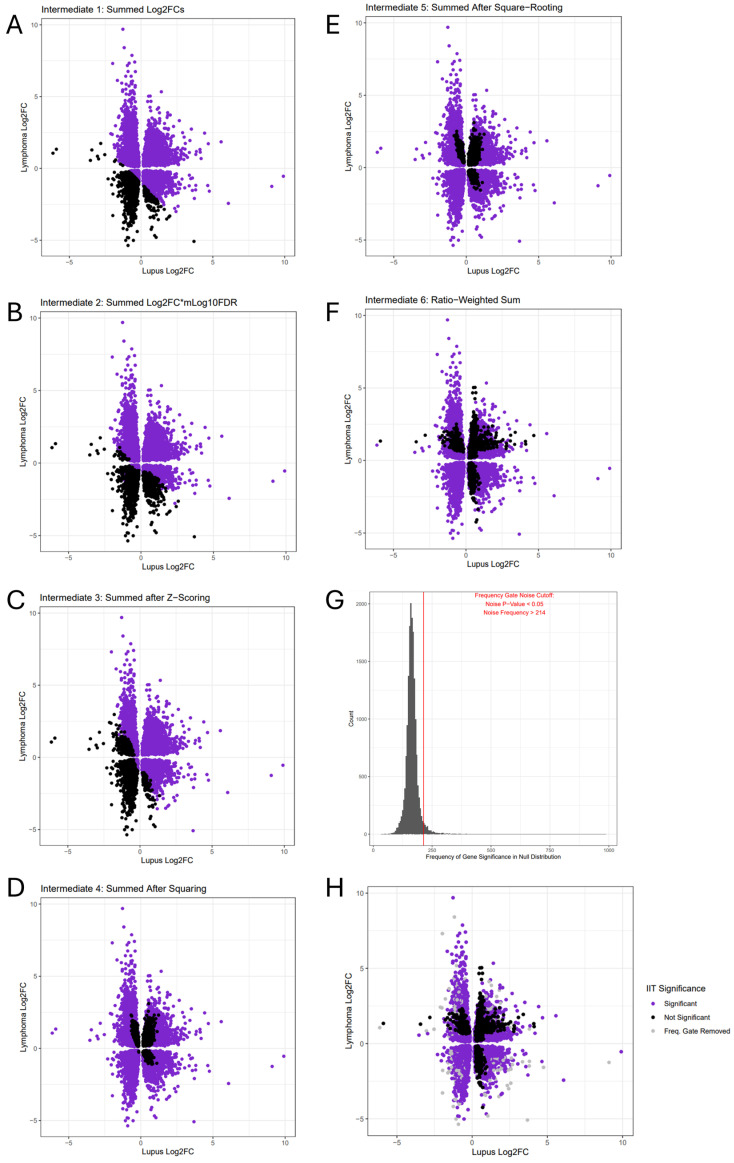
Lupus–Lymphoma IIT Calculation Increases Statistical Stringency through Multiple Filtering Steps. (**A**) Summation of unaltered log2FCs ignores some gene results from three of four quadrants. (**B**) Weighting log2FCs by −log10FDR (mlog10FDR) acknowledges statistical nuance; * represents multiplication in this panel heading. (**C**) Z-scoring log2FC distributions diminishes the bias toward more extreme cancer log2FCs in final summation results. (**D**) Squaring all log2FC values before summation allows gathering of results from all four quadrants. (**E**) Square rooting all values diminishes outlier values. (**F**) Ratio weighting of each summed value includes more results with extreme lupus expression. (**G**) Histogram visualization of gene frequency counts from the IIT-significant portion of the null distribution. The frequency gate threshold, as determined by the *p*-value test, is marked in red on the histogram. (**H**) The differentially expressed genes (DEGs) that were significant in both the lupus vs. healthy and the lymphoma vs. healthy results. The lupus log_2_FCs are along the *x*-axis, while lymphoma log_2_FCs are on the *y*-axis, with each dot representing one gene. Genes with significant IIT scores are plotted in purple. Genes not significant based on the null distribution are plotted in black. Genes that were removed by the noise-detecting frequency gate are plotted in gray. Quadrants in the graph are divided by the axes.

The first important design point is that log2FCs are not the metric used to rank DEGs, rather DEGs are ranked by their false-discovery-rate-corrected *p*-value (FDR). The reason is that the statistical complexity of the distribution of individual samples gene read counts is not fully conveyed by the log2FC value. Some instances of more extreme log2FCs have a less significant FDR than expected due to statistical uncertainty in the data, an overlap between the disease read counts and the healthy read counts. To account for this statistical nuance, we first weighted the log2FC of each gene by the −log10FDR (mlog10FDR) value. This −log10FDR weighting gives an advantage to genes with a clearer distinction between the healthy and diseased gene expression levels (Figure 2A,B and Figure S1).

Secondly, when working with cancer and autoimmune data, no assumption can be made that the differentially expressed genes will have the same extremity of expression in both diseases. In this use-case comparing lupus and lymphoma, we observed a more consistent downregulation of the gene expression in lymphoma than in lupus. Consequently, summation of the log2-fold-change values of cancer and autoimmune disease results in substantial bias toward the cancer log2-fold-change values in the overall ranking. We partially accounted for the bias toward the disease with the more extreme gene expression by applying a z-score normalization of log2FCs (Figure 2C). Z-score normalization converts all values into standard deviations from the mean of the log2FC distribution, making it easier to equitably compare between diseases with broader or narrower distribution curves.

Another design consideration we addressed was how to optimally rank genes with various expression profiles all in one list. Gene expression can be extremely upregulated in both diseases, extremely downregulated in both diseases, or upregulated in cancer but downregulated in autoimmune disease, or downregulated in cancer but upregulated in autoimmune disease. Capturing all four expression profiles is incompatible with using a simple summation of the log2FCs since genes with opposite expression in cancer and autoimmune disease cancel each other out when summed, and result in values close to zero. We therefore decided to square all log2FCs, removing the negative values and allowing for comparison of all four expression profiles simultaneously (Figure 2D). To minimize outlier amplification, and return the values to their original scale, we subsequently took the square-root of all squared values (Figure 2E).

One standing issue, even after z-scoring the log2FC distributions before summation, is the remaining bias toward the disease distribution with more extreme gene expression values (in this case, lymphoma). To identify genes that were dysregulated to a similar degree by both diseases, we incorporated an approach we have called ratio weighting. When making all the possible quotients with the cancer and autoimmune gene values before the final summation, C/A and A/C were the possible quotients. Of these quotients, one was always greater than one after the calculation, and the other was always less than one. The more similar the z-scored and squared log2FCs in the quotient were to one another, the closer to one the division product will be. Since we were interested in identifying genes with quotients close to one, we calculated all C/A and A/C quotients, compiled the smaller quotients (division product less than one) into a list, then multiplied the summed gene scores by their small ratios. This gave an advantage to the genes with ratios closest to one (Figure 2F).

We implemented the following two additional strategies to further account for batch effects: (1) generating a null distribution and calculating a rigorous *p*-value from the 18,486,468 data points included in the null distribution and (2) analyzing the frequency of each gene appearing in the significant portion of the null distribution and removing those that appeared the most (*p*-value < 0.05) using a cutoff called a frequency gate. We generated the null distribution by making 1000 permutations of the initial dataset, randomly shuffling sample phenotypes before the analysis, then deriving the final IIT scores for all genes across all permutations and including these with the original gene results. By calculating this null distribution, consisting of ~18.5 million data points, we enabled a much more rigorous *p*-value cutoff. Results with higher statistical noise and lower IIT scores (and presumably less biological relevance) became insignificant when using the null distribution *p*-value as a cutoff. In addition to the null-distribution-derived *p*-value, we applied a frequency gate to control for genes with high statistical noise that could be attributed to batch effects.

When visualizing frequency counts for all the genes in the IIT-significant portion of the null distribution, we found that the average gene frequency count was 205, meaning that the average gene garnered a significant IIT score in 205 permutations (Figure 2A). We performed z-score normalization on the gene frequency counts, then calculated the *p*-value to identify the genes that had significantly high frequency counts, which indicates significant noise. We removed the genes that were more significant than this frequency gate from further analysis to prevent noise from batch effects, with the significantly immune-imbalanced genes found by IIT localized to the outer edges (representing higher log_2_FCs) and non-significant IIT genes near the center (representing lower log_2_FCs), with genes filtered out by the frequency gate scattered throughout (Figure 2G,H), which is what we expected to see. We observed that the genes removed from our results by the frequency gate were randomly dispersed in relation to log2 fold change, which is what we would expect to see when isolating the variables demonstrating statistical noise. The pattern of significant IIT scores being the most common among genes with extreme log2FCs, the trend of non-significant IIT scores prevailing among genes with low log2FCs, and the random distribution of the frequency-gate-removed “noisy” genes throughout the scatterplot augment our confidence that the IIT algorithm is accomplishing what it was designed to do. Similarly, the rigor of this null-distribution and frequency-gate approach increases the robustness of the significant IIT results. We also recognize that some may prefer to impose additional filters to the results, which is possible since they are exported in tabular format.

### 3.2. Lupus vs. Lymphoma B-Cell Comparison Using IIT Algorithm

We began by identifying the appropriate FASTQ files from relevant studies (e.g., cancer and autoimmune disease samples each compared to the healthy controls) that we would process with our IIT algorithm to predict the targetable gene products in the relevant tissue (Figure 1, Supplementary File S3). The IIT algorithm starts with an RNA-seq dataset consisting of samples from an autoimmune disease, cancer, and healthy tissue controls.

To obtain these data, we searched the GEO database for all eligible lupus and lymphoma samples, healthy B-cell control samples originating from the lupus and lymphoma studies, and additionally gleaned healthy B-cell controls from other human RNA-sequencing studies (Table 1, Supplementary File S1). During the screening, we excluded non-human samples, non-primary samples, non-B-cell samples, and other criteria that would negatively bias or affect our results. Our final dataset included 361 human patient samples, with 135 lymphoma samples, 138 lupus samples, and 89 healthy B-cell samples, from 16 studies [31,32,33,34,35,36,37,38,39,40,41,42,43,44,45]. 

**Figure 3:**
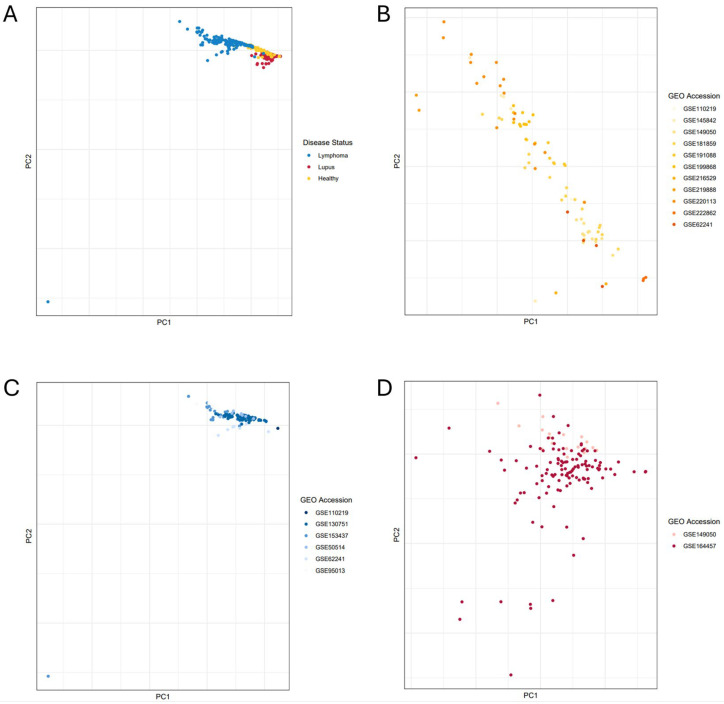
Principal Component Analysis (PCA) of Analyzed Samples. Principal Component Analysis was performed on the count tables of all samples, with lupus samples represented in shades of red, lymphoma samples represented in shades of blue, and healthy samples represented in shades of yellow. (**A**) All samples are colored by disease status. We expected to see samples of the same color grouping together in panel 2A, due to each color representing a distinct disease phenotype (red for lupus, blue for lymphoma, and yellow for healthy). (**B**) Healthy samples colored by the study of origin. (**C**) Lymphoma samples were colored by their study of origin. (**D**) Lupus samples colored by the study of origin.

After identifying the FASTQ files for the relevant human samples, the sequencing reads were trimmed, mapped, and quantified against the human reference transcriptome. Transcript-level read counts were aggregated to calculate the gene read counts. We applied a z-score normalization to the gene read count table (by gene) to avoid amplifying the impact of genes with high read counts and then ran a principal component analysis (PCA) to observe the relationship between samples and evaluate their similarity (Figure 3, Supplementary Figure S2). We found that the samples from different disease statuses (healthy, lupus, or lymphoma patients) generally clustered together (with the exception of one lymphoma sample), and with some overlap between the phenotypic groups (Figure 3A), which is expected given that the samples were obtained from the same cell type with differing phenotypes. Metadata, such as sex, age, ethnicity, and disease diagnosis/stage are incredibly useful but are unfortunately somewhat inconsistent in publicly available datasets. Though we reviewed the literature and sample metadata to collect all the published sample metrics and attributes, many samples were not published with metadata beyond cell type and overall disease category. We visualized sex, age, ethnicity, and reported disease diagnosis/stage (Supplementary Figure S2). Our finding was that samples did not appear to be grouped by age, ethnicity, or sex. Additional studies with metadata are needed to fully evaluate this preliminary finding due to the large quantity of missing metadata in the current dataset. We did note when visualizing the reported lymphoma diagnosis/stage that the diffuse large B-cell lymphoma (DLBCL) samples tended to cluster together, with follicular lymphoma (FL) samples clustering slightly to the side (Supplementary Figure S2). We next colored the samples by study of origin and observed that the samples from each disease/control group were interspersed from the multiple sequencing projects, suggesting a degree of similarity among samples from the same phenotypic groups, with relatively few batch effects among studies or phenotypes (Figure 3B–D, Supplementary Figure S2). These files were then assessed for differential gene expression using the Automated Reproducible Modular workflow for preprocessing and differential analysis of RNA-seq data (ARMOR) [51,52], making two comparisons as follows: lupus vs. healthy and lymphoma vs. healthy. We found ~10,000 genes that displayed significant differential expression in the lupus vs. healthy analysis (Supplementary File S5) and ~16,000 significant genes in the lymphoma vs. healthy analysis (Supplementary File S6). 

After computing the DEGs for the two comparisons (lupus vs. healthy and lymphoma vs. healthy), we applied the IIT algorithm to find genes that have statistically significant differential expression in both comparisons. Briefly, the IIT algorithm includes the following steps: (1) for each of the input DEG sets, the log_2_-fold-change values (log2FCs) are multiplied by their corresponding −log10FDR-adjusted *p*-values to weight the expression of each gene by its statistical significance. Next, the weighted scores for each comparison are z-score normalized prior to taking the square root of the squared normalized value (to eliminate negative values and reduce outliers). These intermediate scores, which still included genes expressing opposite directions, are then summed and normalized using the smaller ratio of either the intermediate score of the lupus vs. healthy comparison or the intermediate score of the lymphoma vs. healthy comparison to generate a final immune imbalance score. To determine the significance of our results and minimize potential batch effects, we calculated a null distribution for these final immune imbalance scores by performing 1000 permutations of the original DEG analysis (Supplementary Files S7–S10), by randomly assigning samples to disease groups in each permutation and calculating IIT values for all genes, resulting in 18,468,000 permuted IIT final scores (Supplementary File S11). This null distribution aids with controlling for batch effects by assigning statistical significance only to IIT scores which have a strong enough signal to rise above the statistical noise. We then z-score normalized the IIT null distribution to assign significant *p*-values (*p* < 0.05) to the highest IIT scores on the upper tail of the null distribution, where the most intensely immune-imbalanced results should be located. We then performed multiple hypothesis testing corrections using the stringent Bonferroni method. Following the assignment of IIT score *p*-values based on the null distribution, we conducted one additional test to remove noise. Using the ~18 million data points in the null distribution, we tallied up how many times a gene was considered IIT significant in 1 of the 1000 data permutations to remove genes with abundant frequency counts from the IIT-significant portion of the null distribution.

We then assessed the DEGs from the lupus vs. healthy and lymphoma vs. healthy differential expression analyses using the Immune Imbalance Transcriptomics (IIT) algorithm. Out of the initial overlapping significantly differentially expressed pool of 7143 genes, we observed that 5363 had statistically significant immune imbalance (Bonferroni *p*-value < 0.05; Supplementary File S12). Following application of the frequency gate as described above, 5137 significant genes were left for consideration (Supplementary File S13). When we visualized overlapping gene results from the lupus and lymphoma differential gene comparisons using log2FCs as the axis scales and one scatter-point per gene, we observed that the overlapping genes fell into four quadrants. Functionally, these gene profiles could be categorized into four quadrants. For clarity, we labeled these quadrants as “cancer” (lymphoma in this use-case) and “autoimmune disease” (lupus in this use-case), knowing that this algorithm can be applied to different cancer–autoimmune pairings. The four quadrants are as follows: upregulated in both lymphoma (Cancer) and lupus (Autoimmune disease; Quadrant I, C+A+), downregulated in both lymphoma and lupus (Quadrant III, C−A−), upregulated in lymphoma but downregulated in lupus (Quadrant II, C+A−), and downregulated in lymphoma but upregulated in lupus as compared to the healthy samples (Quadrant IV, C−A+). The top four immune-imbalanced genes from each quadrant are represented in Table 2. Among the top four IIT genes per quadrant are several gene products that have previously been shown in other studies to be involved in lupus and/or lymphoma, including Membrane Associated Ring-CH-Type Finger 8 (*MARCH8*; Quad IV) [74].

**Figure 4:**
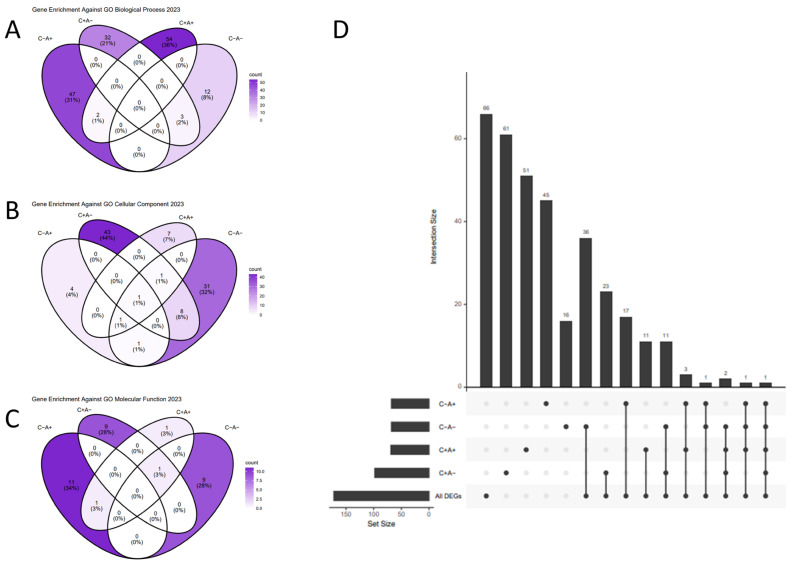
Lupus–Lymphoma IIT Gene Quadrants and GO Enrichment. (**A**) Gene Ontology (GO) term enrichment, by quadrant, against the Biological Process GO library. (**B**) Gene Ontology (GO) term enrichment, by quadrant, against the Cellular Component GO library. (**C**) Gene Ontology (GO) term enrichment, by quadrant, against the Molecular Function GO library. (**D**) Upset chart (an alternative to a five-way Venn diagram) showing the overlapping GO Results from all included GO libraries between individual quadrant enrichment lists and the enrichment list of the entire significant gene dataset.

To identify the relevant functional terms and signaling cascades in the significant IIT results, we ran separate Gene Ontology (GO) and intracellular pathway hypergeometric enrichment analyses on the significant IIT genes using the EnrichR package (version 3.2) [62]. We retrieved all GO terms individually for the significant IIT genes, compiled them for easy reference (Supplementary File S14), and utilized a text search to determine how many GO term descriptions mentioned “immune” or “immunity” for each gene. We ran GO term enrichments on the genes in each quadrant (Figure 4A–C, Supplementary File S15), and an additional hypergeometric enrichment on the entire significant IIT gene set (Table 3, Supplementary File S15). We were somewhat surprised to observe relatively few overlapping terms between the quadrants in these GO enrichment results (Figure 4A–C), suggesting that genes in different quadrants potentially play unique functional roles. Interestingly, we also found many quadrant-specific results overlapping with the GO enrichment terms obtained from enriching all the significant IIT genes together (Figure 4D).

We found 864 significantly enriched signaling pathways from the four databases used in the analysis when looking at analytical results from each quadrant and all genes combined (redundancy exists between quadrant results; Table 4, Supplementary File S16, Supplementary Figure S3). We noted 4 pathways with immune-related function among the top 10 significantly enriched, including “Neutrophil Degranulation”, “Innate Immune System”, “Immune System”, and “Adaptive Immune System”. This validation is an important step in addressing the potential batch effects by evaluating the biological relevance of our results. The presence of these significant immune-related terms among our top results of significantly enriched pathways involved in immune function supports our hypothesis that our IIT algorithm can identify genes relevant to immune function. It also introduces the possibility that other significant IIT genes with no currently known involvement in immune effector function and immune signaling may play uncharacterized roles in the immune function of B cells.

We also observed multiple pathways relating to cell cycle regulation and cell division in our top ten enriched pathway results, including the control of the cell cycle such as “Transcriptional Regulation By TP53“; as well as pathways involved in increasing transcription and translation such as “Metabolism Of RNA”, “Metabolism Of Proteins”, and “Translation”, suggesting heightened activity that could be attributed to cell division. These findings augment our confidence in these results due to the well-known high rate of cell division in lupus auto-reactive B cells and the high rate of aggressive division in B-cell lymphoma. This serves as an internal control that the IIT algorithm finds biologically relevant genes to B cell function in the context of lupus and lymphoma pathology.

Following the pathway enrichment analysis, we analyzed the list of significant immune-imbalanced genes to identify therapeutic targets for potential drug repurposing. Using the OpenTargets database [73] and an adaptation of the Pathway2Targets algorithm [75], we predicted targets that could be utilized against lupus or lymphoma. A promising outcome of our target prediction analysis is that we found 344 (out of 5137 significant immune-imbalanced genes) with known drugs that are either approved or in development for at least one indication (Table 5, Supplementary Files S17 and S18). Lupus currently has 172 targets with known drugs, 48 of which are significantly immune-imbalanced in this study. Lymphoma currently has 437 targets with drugs in testing, and 151 of these targets appear on our significantly immune-imbalanced list. These known lupus and lymphoma drug targets in our significant immune-imbalanced results provide further veracity to the biological relevance of the IIT gene results to lupus and lymphoma treatment. Additionally, we found 296 novel targets for lupus and 193 novel targets for lymphoma using IIT, which may be useful in the future. We also noted 37 known targets shared by both lupus and lymphoma, suggesting that these drugs may be particularly useful to patients fighting both illnesses.

## 4. Discussion

In this work, we identified ~16,000 genes differentially expressed between the lymphoma and healthy B cells and ~10,000 genes between the lupus B cells and healthy B cells, and the top results are biologically relevant to the pathology of these B-cell diseases. Initial PCA analysis of this dataset showed that the samples were grouped by disease as expected (Figure 3A), and that the samples from individual studies were mixed within the disease groups, indicating minimal batch effects (Figure 3B–D). We did not correct for batch effects at the study level, due to most commonly accepted batch effect correction algorithms relying on the presence of both case and control samples in each dataset. Many of our datasets contain only one phenotype (just healthy samples, etc.), with a nominal percentage of studies containing both healthy and disease samples, and none containing all three phenotypes being evaluated. Using batch effect correction on a portion of the studies while leaving other studies untouched before performing analysis was not ideal. As such, we decided to validate our results by checking biological relevance and correct for statistical noise potentially resulting from batch effects downstream using an established and a novel strategy.

We used downstream validation and significance measurements to remove genes with high batch effects and validate the biological relevance of our IIT results. As described in the results, we noted that generating a null distribution with ~18.5 million gene significance scores removed many of the previously significant scores from the immune-imbalanced gene list. We noted that this expanded the spread of black dots in the middle of the log2FC scatter plots, which dots are representative of less extremely dysregulated genes. In addition, we observed the effectiveness of the frequency gate at removing genes with significant statistical noise in their read counts, as measured by the number of times each gene appeared in the significant tail of the null distribution. We noted that these genes removed by the frequency gate were randomly distributed throughout the gene results with no evident correlation to the log2FC, which strengthens our confidence that the algorithm is removing the genes displaying evidence of statistical noise. The random distribution of these genes is what we expected to find.

We hypothesized that the IIT algorithm would isolate genes related to inflammation and immune function. We used the DEG lists from these two comparisons as a use case to demonstrate the ability to identify robust and biologically relevant mechanisms in lupus and lymphoma using the significant 5137 gene products identified by the null distribution within our novel Immune Imbalance Transcriptomics (IIT) algorithm, as well as biologically relevant enrichment results and potential therapeutic targets. Previous bioinformatic approaches have investigated the shared transcriptomic mechanisms of disease between an autoimmune disease and a cancer from the same tissue type [25] but have not accounted for potential contrasting mechanisms, a novel attribute of IIT. Following the evaluation of IIT significance of each gene, we performed a rigorous validation using multiple methods to discover the degree of biological relevance of our results.

Though inflammation is classically activated by infection or injury, other causes exist, including underlying tissue malfunction, which induces para-inflammation as the body attempts to return to homeostasis [76]. Para-inflammation has previously been implicated as the cause of chronic inflammatory conditions, including cancer and autoimmune disease [76]. The immune imbalance algorithm is designed to analyze a tissue-specific pairing of one cancer and one autoimmune disease, along with healthy tissue controls, for the purpose of identifying genes which are extremely regulated to an equal degree by both the cancer and the autoimmune disease, including those genes that effect (or are affected by) inflammation. 

We found relatively few overlapping GO terms between the quadrants, which seemingly indicates independent mechanisms belonging to each expression quadrant. This gives support to the approach of leveraging genes from different quadrants to accomplish diverse treatment results, highlighting the importance of characterizing immune imbalanced genes from all quadrants. 

Though GO term results do not specifically lend support to the immune/inflammatory functional identity of the IIT genes, the significantly enriched pathway results made a strong case for their role in immune/inflammatory response. “Cellular Responses to stress” usually involves inflammation, so 5 of the top 10 pathway results describe inflammatory or immune-related processes (e.g., “Neutrophil Degranulation”, “Immune System”, “Adaptive Immune System”, and “Innate Immune System”). This confirms our hypothesis that comparison using IIT can bring to the forefront genes relevant to inflammation and immune function.

There is growing evidence that cancer and autoimmune diseases can have a causal or correlative relationship, with the development of one creating a greater risk for the development of the other in the same patient [19,25,77,78,79,80,81,82,83]. The rise in co-occurrence risk suggests that the two diseases likely share some underlying causes and common mechanisms, such as chronic inflammation [83]. The rising occurrence of chronic inflammatory diseases and their co-incidence justifies a new research approach as follows: finding, characterizing, and modifying the shared underlying disease pathologic mechanisms. This study exemplifies this approach in our identification of IIT genes from quadrants I and III, which focus on similarly upregulated and similarly downregulated gene mechanisms, respectively. Instead of focusing solely on the similar mechanisms of pathology, treatment success may also be found by incorporating complementary mechanisms between cancer and autoimmune disease, such as the IIT genes of quadrants II and IV (C+A− and C−A+, respectively). A potential extension of current immunotherapeutic treatments is harnessing the power of these contrasting immune imbalance mechanisms, such as genes described in this study, to pull patients toward homeostasis. For example, a gene upregulated in lupus B cells may provide clues about how to modulate the immune microenvironment of B-cell lymphomas; likewise, an immuno-modulatory gene upregulated by B-cell lymphoma could possess the power to dampen the rampant inflammation of lupus.

Perhaps the most easily leverageable gene mechanisms presented in this study belong to a cohort of IIT genes with known drug–protein interactions recorded in the OpenTargets database (Table 5). Represented in this cohort are genes from all four quadrants, indicating possibilities for immediate testing. We would like to note that these therapeutics are likely to have substantial effects on the cell biology of tested systems; however, results should be critically evaluated before a trial to ensure that a researcher does not apply an agonist where an antagonist is needed. The genes identified by IIT that could be evaluated for drug repurposing include Complement C3 (*C3*; Quad II), C-X-C Motif Chemokine Receptor 4 (*CXCR4*; Quad III), Mitogen-Activated Protein Kinase Kinase Kinase 1 (*MAP3K1*; Quad IV), And Cellular Communication Network Factor 2 (*CCN2*; Quad I).

Though drug repurposing is not always possible due to the lack of viable drug candidates, drug discovery, or other methods of gene targeting must be undertaken. Many experimental methods for the in vivo activation or inhibition of gene expression are currently under study and consideration, including gene delivery by engineered viruses [84] and Clustered Regularly Interspaced Short Palindromic Repeats (CRISPR) methods (e.g., CRISPR activation (CRISPRa), CRISPR inhibition (CRISPRi), etc.) in pathogenic cells to increase or decrease gene expression [85]. A current limitation in the field of in vivo gene editing is that it is difficult to target the correct cells while minimizing off-target effects. However, we anticipate that, with further development, such approaches could enable in vivo gene therapy to treat immune-related diseases including cancer and autoimmunity using immune-imbalanced targets. These approaches may be viable in the future for expression editing of even genes with known drug–protein interactions. Good examples of the 37 significantly immune-imbalanced genes we found already in use as drug targets for both lupus and lymphoma which could be targeted using gene therapy are CD3g Molecule (*CD3G*; C+A− quadrant), Janus Kinase 1 (*JAK1*; C−A+ quadrant), and Prostaglandin-Endoperoxide Synthase 1 (*PTGS1*; C−A− quadrant).

Our intent in conducting this study was to leverage gene products brought up in this analysis as leads on disease biomarkers, treatments, and potentially targetable mechanistic markers. The following are examples of IIT genes we can see being utilized in the treatment or monitoring of patients.

A subset of potential agonistic targets could benefit patients due to their significant downregulation in both lupus and lymphoma B cells. Each is a good candidate for upregulation/agonistic treatment in pathogenic lupus and lymphoma cells due to their known involvement in lupus and lymphoma progression via under-expression, although care should be taken to minimize adverse events with these potential targets. TSC22 Domain Family Member 3 (*TSC22D3*, C−A− quadrant) is a proapoptotic gene in some cell types and is positively regulated by Estrogen-Related Receptor-β (ESRRB) [86]. Previous studies have shown that ESRRB repression in acute lymphoblastic leukemias, which are closely related to B-cell lymphomas, contributes to treatment resistance. This suggests that activating *TSC22D3* would improve the success of malignant B-cell treatment [86]. Kruppel Like Factor 6 (KLF6; C−A− quadrant) is a key part of recruiting macrophages to the inflammation site and the immune response subsequent success, which is lacking in cancers, including B-cell lymphomas [87]. These data suggest the potential benefits of an antagonist. Transmembrane Anterior Posterior Transformation 1 (TAPT1, C−A− quadrant), ADP Ribosylation Factor Like GTPase 4A (*ARL4A*, C−A− quadrant), and Proline-Rich Nuclear Receptor Coactivator 1 (PNRC1, C−A− quadrant) are novel results which, as underlying mechanistic genes shared by lupus and lymphoma, could be leveraged as possible therapeutic targets.

Among our novel immune-imbalanced genes are disease target candidates for antagonists in both lupus and lymphoma patient treatments. However, very little is known about many of these significant IIT genes (located in Quadrant I) in the context of lupus and lymphoma B cells. Clinical analyses have shown that tumors expressing increased levels of NFE2-Like bZIP Transcription Factor 3 (NFE2L3, C+A+ quadrant) indicated a worse prognosis [88], making it an ideal candidate for future knockdown studies. Parathymosin (*PTMS*, C+A+ quadrant) and Plexin A1 (*PLXNA1*, C+A+ quadrant) are both novel results with potential to be targeted in agonistic treatment for both diseases.

Several immune imbalance genes in this analysis are good candidates for therapeutic agonists. OTU Deubiquitinase 1 (OTUD1; C−A+ quadrant) has been shown to repress type-I interferon-mediated disease, which includes lupus, in vivo [89] and to have loss-of-function in systemic lupus erythematosus [90], suggesting the promise of treatments that increase the activity of OTUD1 in lupus B cells. Membrane Associated Ring-CH-Type Finger 8 (MARCH8; C−A+ quadrant) plays a regulatory role in lymphoma by downregulating CD98, which promotes proliferation and cell division in lymphomas [74]. These promising targets for gene-upregulation treatment present new possibilities for patients.

Additionally, many of the top immune-imbalanced genes could be potential targets for antagonists. Complement C1qB Chain (*C1QB*, C+A− quadrant), Increased *C1QB* expression decreases the effectiveness of combined Ibrutinib and Venetoclax in patients with Mantle-Cell lymphoma (a B-cell lymphoma) and is associated with a worse prognosis [91]. Insulin-Like Growth Factor Binding Protein 2 (*IGFBP2*, C+A− quadrant), Targeting and neutralizing IGFBP2 (C+A− quadrant) as a lupus nephritis biomarker shows potential as a new option in treating patients with lupus nephritis, an advanced complication of SLE targeting the kidneys with few workable treatments [92,93]. Transcobalamin 2 (*TCN2*, C+A− quadrant), *TCN2* may be a predictive biomarker in primary large B-cell lymphoma of immune-privileged sites, making it a good early target to prevent metastasis [94]. Treatment-induced antagonism or knockdown of the gene products in these cases could also improve patient outcomes. Transmembrane Anterior Posterior Transformation 1 (*TAPT1*, C−A+ quadrant) and Proline-Rich Nuclear Receptor Coactivator 1 (*PNRC1*, C−A+ quadrant) are novel immune imbalance results with no known mechanisms and with promising potential to change pathogenic properties of lupus and lymphoma B cells, which justifies future experimentation.

While our IIT results can be useful, it is important to explicitly recognize at least some of the inherent limitations of this study. As demonstrated in Table 1, this work was accomplished using publicly available datasets containing either lupus, lymphoma, or healthy B cell RNA sequencing samples. There is a notable lack of consistency in the metadata provided by each of the original projects, with some projects providing great detail and others providing none beyond the basic diagnostic phenotype and sample tissue type. Though this made in-depth comparison impossible for some metadata categories, we screened data very carefully to ensure the highest quality of samples based on the information provided. We continue to advocate continued compliance with the findable, accessible, interoperable, and reusable (FAIR) data guidelines to support the success of future combined analyses such as applications of IIT.

We recognize that not all types of B-cell lymphoma were studied since our sample pool was limited to the publicly available datasets on the Gene Expression Omnibus (GEO). Diffuse large B-cell lymphoma was prominent in these samples due to it being one of the most common and aggressive subtypes of B-cell non-Hodgkin’s lymphoma. As such, we expect that these results will be especially applicable to diffuse large B-cell lymphoma, with limited relevance being likely for the other lymphoma subtypes, due to fewer contributed samples. 

Another potential area of limitation arises from including multiple sequencing projects in the same differential gene expression analysis. As no studies contained the representative samples we required, we decided to combine multiple studies and indirectly account for batch effects within the algorithm itself. The gene-specific batch effects are particularly important, which would boost or lower read counts for a specific gene. 

In summary, our novel algorithm, Immune Imbalance Transcriptomics (IIT), identifies the shared and unique transcriptional mechanisms between cancer and autoimmunity in the same tissue (or cell) type. The biological use case for this algorithm comparing lupus and lymphoma B cells is an important step forward and suggests that an algorithmic approach may enable the ability to glean additional mechanistic insights and continue to advance the state of diagnostics and treatment for cancer and autoimmune patients alike. The IIT approach allows for a more robust consideration of the effects of immune-related gene products on identifying therapeutic targets that can help to return the lupus and lymphoma phenotypes to a more immune-balanced, non-inflamed homeostasis. We anticipate that the identified mechanisms and targets will aid future efforts to develop improved therapeutics for both lupus and lymphoma. As one of the first reproducible methods for comparing cancer and autoimmune transcriptional effects, our IIT algorithm potentially provides a novel approach to a better understanding of the mechanisms of lupus and lymphoma disease and how to target these mechanisms without upsetting the immune balance. We expect that this approach can improve the quality of life for patients by providing effective treatment options with fewer and less severe side effects.

## 5. Patents

N.R.-S. and B.E.P. are filing a patent on the Immune Imbalance Transcriptomics algorithm.

## Figures and Tables

**Table 1:** Sample Metadata Summary.

Sample Phenotype	Type of Sequencing Reads	GEO Identifier	Number of Relevant Samples Included in Current Study
large B-cell lymphoma	paired end	GSE153437 [31]	25
follicular lymphoma	paired end	* GSE62241 [32,33]	10
diffuse large B-cell lymphoma	paired end	GSE95013 [34]	29
B-cell lymphoma	single end	* GSE110219 [35]	1
diffuse large B-cell lymphoma	paired end	GSE130751 [36]	63
diffuse large B-cell lymphoma	paired end	GSE50514 [37]	7
lupus B-cells	single end	* GSE149050 [38]	18
lupus B-cells	paired end	GSE164457 [39]	120
healthy B-cells	paired end	GSE145842 [40]	6
healthy B-cells	single end	* GSE149050 [38]	14
healthy B-cells	paired end	GSE181859 [41]	20
healthy B-cells	paired end	* GSE62241 [32,33]	4
healthy B-cells	single end	* GSE110219 [35]	1
healthy B-cells	paired end	GSE191088 [42]	6
healthy B-cells	paired end	GSE199868 (currently unpublished)	13
healthy B-cells	paired end	GSE216529 [43]	2
healthy B-cells	single end	GSE219888 [44]	2
healthy B-cells	paired end	GSE220113 [45]	17
healthy B-cells	single end	GSE222862 (currently unpublished)	3

* GSE62241, GSE110219, and GSE149050 contain both disease samples and healthy controls. For clarity, they are each included in the table twice.

**Table 2:** Top Four Significantly Immune-Imbalanced Genes from Each IIT Quadrant.

	Gene Symbol	Lupus * log2FC	Lupus * FDR	Lymphoma ** log2FC	Lymphoma ** FDR	IIT Score	IIT Corrected *p*-Value ***
C+A+ Quad I	*TPM2*	1.85	3.25 × 10^−8^	3.88	1.65 × 10^−25^	21.81	0
	*PTMS*	2.03	2.05 × 10^−10^	3.72	8.08 × 10^−27^	20.21	0
	*PLXNA1*	1.11	3.14 × 10^−12^	2.75	1.04 × 10^−34^	19.35	0
	*SIX5*	1.72	5.53 × 10^−12^	2.79	7.36 × 10^−34^	18.76	0
C+A− Quad II	*LGALS3BP*	−1.57	3.57 × 10^−10^	3.55	3.62 × 10^−36^	27.08	0
	*VSIG4*	−1.65	5.81 × 10^−8^	6.13	4.83 × 10^−29^	17.62	0
	*FKBP5*	−1.33	1.77 × 10^−9^	2.25	8.87 × 10^−36^	16.57	0
	*ARMCX1*	−1.19	8.57 × 10^−7^	2.87	3.10 × 10^−22^	12.87	0
C−A− Quad III	*MED30*	−0.895	1.54 × 10^−12^	−1.46	5.07 × 10^−41^	19.64	0
	*ING3*	−0.983	1.58 × 10^−17^	−1.63	3.96 × 10^−40^	18.15	0
	*OSER1*	−0.752	6.09 × 10^−14^	−1.6	8.45 × 10^−50^	17.44	0
	*PLD4*	−1.05	2.23 × 10^−10^	−3.5	6.43 × 10^−26^	17.12	0
C−A+ Quad IV	*PNRC1*	1.21	5.07 × 10^−16^	−2.11	1.16 × 10^−43^	28.32	0
	*SLC12A6*	1.04	6.91 × 10^−15^	−2.46	3.48 × 10^−37^	21.8	0
	*OTUD1*	1.47	7.92 × 10^−18^	−2.24	5.46 × 10^−37^	21.37	0
	*MARCH8*	0.932	6.91 × 10^−15^	−1.86	5.35 × 10^−40^	19.8	0

* Systemic Lupus Erythematosus; ** B-cell Non-Hodgkin’s Lymphoma; *** Bonferroni-corrected; Abbreviations: Tropomyosin 2 (TPM2), Parathymosin (*PTMS*), Plexin A1 (*PLXNA1*), SIX Homeobox 5 (*SIX5*), Galectin 3 Binding Protein (*LGALS3BP*), V-Set And Immunoglobulin Domain Containing 4 (*VSIG4*), FKBP Prolyl Isomerase 5 (*FKBP5*), Armadillo Repeat Containing X-Linked 1 (*ARMCX1*), Mediator Complex Subunit 30 (*MED30*), Inhibitor Of Growth Family Member 3 (*ING3*), Oxidative Stress Responsive Serine Rich 1 (*OSER1*), Phospholipase D Family Member 4 (*PLD4*), Proline Rich Nuclear Receptor Coactivator 1 (*PNRC1*), Solute Carrier Family 12 Member 6 (*SLC12A6*), OTU Deubiquitinase 1 (*OTUD1*), Membrane Associated Ring-CH-Type Finger 8 (*MARCH8*).

**Table 3:** Top Five GO Terms from Enrichment of All Significant IIT Genes.

Term	Overlap	Bonferroni *p*-Value	Odds Ratio	Combined Score	GO DAG *
RNA Binding (GO:0003723)	542/1411	7.08 × 10^−26^	1.92	124.82	Molecular Function
Cytoplasmic Translation (GO:0002181)	42/93	3.05 × 10^−16^	8.33	366.05	Biological Process
RNA Binding (GO:0003723)	193/1411	1.36 × 10^−11^	1.95	61.83	Molecular Function
Proton Motive Force-Driven ATP Synthesis (GO:0015986)	26/60	4.07 × 10^−10^	8.99	268.95	Biological Process
RNA Binding (GO:0003723)	209/1411	5.27 × 10^−10^	1.82	50.73	Molecular Function

* GO DAG: Gene Ontology Directed Acyclic Graph.

**Table 4:** Top 10 Enriched Pathways against All Significant IIT Genes.

Pathway	Overlap	FDR *p*-Value	Odds Ratio	Combined Score	Database
Neutrophil Degranulation	197/468	1.54 × 10^−15^	2.17	74.13	Reactome
Innate Immune System	372/1035	1.06 × 10^−14^	1.69	54.48	Reactome
Immune System	636/1943	2.44 × 10^−14^	1.49	46.55	Reactome
Metabolism Of RNA	253/666	2.96 × 10^−13^	1.83	52.94	Reactome
Cellular Responses To Stress	268/722	1.22 × 10^−12^	1.77	48.52	Reactome
Cellular Responses To Stimuli	272/736	1.46 × 10^−12^	1.76	47.88	Reactome
Metabolism Of Proteins	608/1890	3.59 × 10^−12^	1.44	37.98	Reactome
Transcriptional Regulation By TP53 *	145/354	9.17 × 10^−11^	2.06	47.65	Reactome
Translation	120/281	1.77 × 10^−10^	2.21	49.64	Reactome
Adaptive Immune System	261/733	3.45 × 10^−10^	1.65	36.00	Reactome

* Abbreviations: Tumor Protein 53 (TP53).

**Table 5:** Top-Scoring Immune-Imbalanced Targets with Known Drugs.

Gene Symbol	Quadrant	Immune Imbalance Score	IIT Corrected *p*-Value *	Number Unique Drugs	Number Approved Drugs	Weighted Target Score
*MAP3K1*	Quad IV	16.47	0	1	0	698
*NR1H2*	Quad III	15.24	0	5	0	186
*KCNQ1*	Quad IV	12.96	0	8	1	662
*GABBR1*	Quad IV	11.8	0	7	1	320
*PIKFYVE*	Quad IV	11.79	0	2	0	123
*SIRT1*	Quad IV	10.86	0	1	0	1219
*PTGER4*	Quad III	9.64	0	4	1	438
*CCN2*	Quad I	9.25	0	1	0	927.5
*CXCL10*	Quad II	8.81	0	2	0	1212
*CD3G*	Quad II	8.29	0	11	1	195

* Bonferroni-adjusted *p*-value; Abbreviations: Mitogen-Activated Protein Kinase Kinase Kinase 1 (*MAP3K1*), Nuclear Receptor Subfamily 1 Group H Member 2 (*NR1H2*), Potassium Voltage-Gated Channel Subfamily Q Member 1 (*KCNQ1*), Gamma-Aminobutyric Acid Type B Receptor Subunit 1 (*GABBR1*), Phosphoinositide Kinase, FYVE-Type Zinc Finger Containing (*PIKFYVE*), Sirtuin 1 (*SIRT1*), Prostaglandin E Receptor 4 (*PTGER4*), Cellular Communication Network Factor 2 (*CCN2*), C-X-C Motif Chemokine Ligand 10 (*CXCL10*), CD3g Molecule (*CD3G*).

## Data Availability

The datasets analyzed during the current study are available in the Gene Expression Omnibus (GEO) repository. All GEO accession numbers for included sequencing projects can be found in Table 1, while individual SRA identifiers for included samples can be viewed in Supplementary File S1. All Supplementary Materials are cited within the text and are accessible at https://doi.org/10.5281/zenodo.13771034.

## Supplementary Materials

The following supporting information can be downloaded at https://doi.org/10.5281/zenodo.13771034 (accessed on 16 September 2024), File S1: Metadata for Samples Used in RNA-Seq Analysis; Figure S1: Additional Graphs of Intermediate IIT Steps; Figure S2: Principal Component Analysis (PCA) of All Analyzed Samples by Study; File S2: Immune Imbalance Transcriptomics Algorithm; File S3: Lupus txiMeta Object for Algorithm; File S4: Lymphoma txiMeta Object for Algorithm; File S5: Lupus edgeR Results; File S6: Lymphoma edgeR Results; File S7: Lupus permutation deck; File S8: Lymphoma permutation deck; File S9: EdgeR results for all lupus permutations; File S10: EdgeR results for all lymphoma permutations; File S11: Null distribution with ~18.5 million IIT scores from original analysis and 1000 permutations; File S12: genes that were IIT-significant after null distribution *p*-value determination and removal of noisy genes using frequency gate; File S13: All significant IIT genes, including those excluded by the frequency gate; File S14: All GO terms by gene for easy reference; File S15: GO enrichment results; File S16: pathway enrichment results; Figure S3: Pathway Enrichment Venn Diagrams; File S17: Drugs Known to interact with significant immune-imbalanced genes; File S18: Immune Imbalance Genes which are also known drug targets.
